# Endoscopic endonasal resection of olfactory tract hamartoma for pediatric epilepsy

**DOI:** 10.1007/s00381-024-06595-2

**Published:** 2024-09-02

**Authors:** Adam J. Kundishora, Benjamin C. Reeves, David K. Lerner, Phillip B. Storm, Marisa S. Prelack, James N. Palmer, Nithin D. Adappa, Benjamin C. Kennedy

**Affiliations:** 1 Department of Neurosurgery, Children’s Hospital of Philadelphia, Philadelphia, PA USA; 2 Department of Otorhinolaryngology-Head and Neck Surgery, Perelman School of Medicine, University of Pennsylvania, Philadelphia, PA USA; 3 Department of Neurology, Children’s Hospital of Philadelphia, Philadelphia, PA USA; 4 Department of Neurosurgery, University of Pennsylvania, Philadelphia, PA USA

**Keywords:** Hamartoma, Pediatric, Olfactory bulb, Epilepsy, Endoscopic

## Abstract

**Background:**

Non-hypothalamic glioneural hamartomas are rare entities known to cause medically refractory epilepsy. Olfactory bulb hamartomas, in particular, are exceptionally rare.

**Methods:**

We describe a case of an olfactory bulb hamartoma that was surgically resected at our institution. We also performed a literature review of all glioneural hamartomas and discuss the clinical presentation, diagnosis, and management of these lesions.

**Results:**

Herein, we present the unusual case of a typically developing 17-year-old boy with a near life-long history of drug-resistant epilepsy, found to have a 0.8 × 1.0 cm right olfactory bulb hamartoma. Endoscopic endonasal trans-cribriform resection of the lesion led to seizure freedom in the 6-month follow-up period (Engel class 1 outcome). Comprehensive literature review revealed only one other sporadic case, which was also successfully treated with total surgical resection.

**Conclusions:**

Our case of an olfactory bulb hamartoma adds to the limited literature currently available, illustrating key clinical characteristics of these exceedingly rare lesions and outlining an effective, minimally invasive, and low-morbidity treatment strategy.

**Supplementary Information:**

The online version contains supplementary material available at 10.1007/s00381-024-06595-2.

## Introduction

Central nervous system (CNS) hamartomas are benign glioneuronal malformations composed of disorganized mature neurons and glial cells [1]. While hypothalamic hamartomas and the tubers of tuberous sclerosis are well-characterized hamartomatous entities in pediatric epilepsy [2], hamartomas elsewhere in the brain, such as within the lateral or fourth ventricle [3, 4], frontal or temporal lobe [5, 6], subependymal space [7], cerebellum [8], brainstem [9], olfactory bulb [10, 11], or optic nerve [12], are comparatively very rare. In particular, sporadic hamartomas of the olfactory bulb or tract are exceptionally rare, with only one non-syndromic case previously reported in the literature [10, 11]. Focal symptoms vary with the location of the lesion, but epilepsy is the most common manifestation of all glioneural hamartomas, sometimes leading to devastating neurodevelopmental morbidity [6, 13–16]. Treatment of hamartomas primarily relies on surgical resection or ablation which can alleviate associated seizures. Thus, with early recognition and timely surgical intervention, the neurological sequalae often seen with prolonged intractable epilepsy can be prevented, abated, or even reversed [16].

Here, we describe a case in which endoscopic endonasal trans-cribriform (EETC) surgical resection of an olfactory tract hamartoma resolved seizures in an otherwise healthy boy with a 14-year history of drug-resistant epilepsy.

## Illustrative case

A 17-year-old boy with no family history of seizures presented with a 13-year history of epilepsy. His first seizures began at 4 years old, during which time he had three seizures in less than a week. These events were not associated with any head injury, serious illness, or fever. Seizures were focal with impaired awareness, consisting of behavioral arrest, leaning to the left, and left arm hypomotor semiology, generally lasting between 30 and 120 s. Seizure control on oxcarbazepine was variable over the next 12 years, but eventually he progressed to having near monthly seizures despite further increases in dosage and additional treatment with levetiracetam. He otherwise developed normally with no overt neurologic deficits, but upon questioning reported a subjective mild anosmia compared to others.

**Fig. 1:**
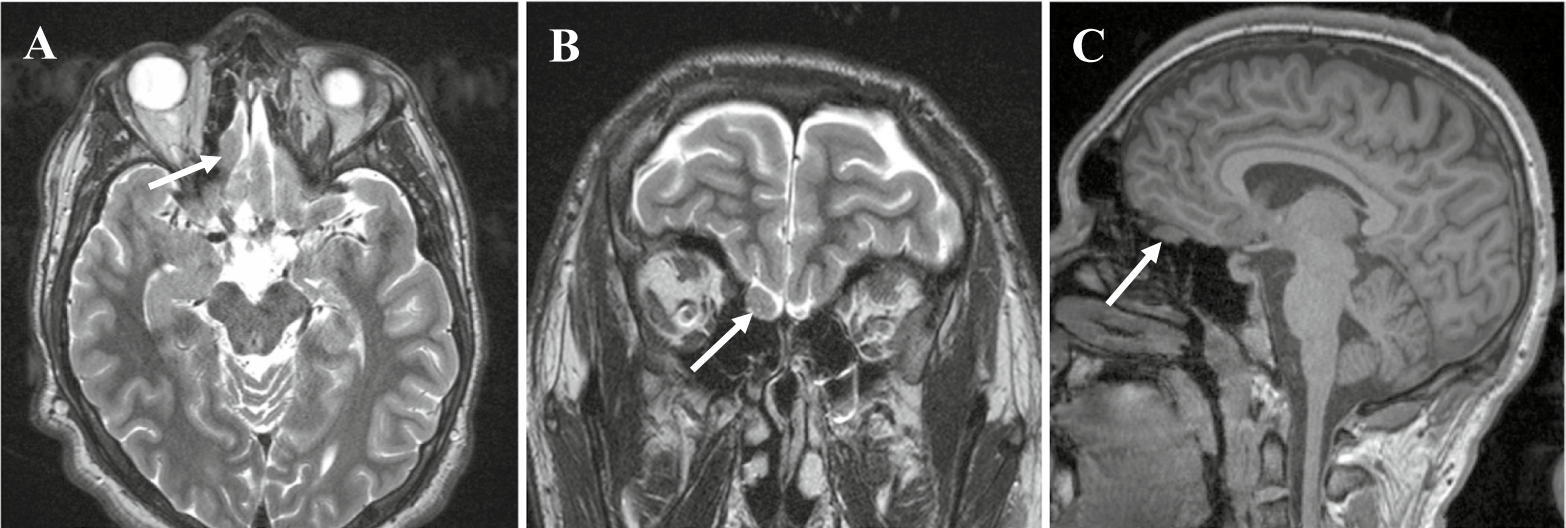
Axial (**A**) and coronal (**B**) preoperative T2-weighted and sagittal preoperative T1-weighted non-contrast (**C**) magnetic resonance images (MRI) demonstrating 0.8 × 1.0 cm ovoid mass in right olfactory tract (white arrows)

He underwent a negative genetic workup. Brain magnetic resonance imaging (MRI) demonstrated a 1.0 × 0.8 cm fusiform lesion arising from the right olfactory tract with mild local mass effect on the overlying orbitofrontal gyrus and gyrus rectus without edema (Fig. 1A–C). Electroencephalogram (EEG) demonstrated occasional right frontopolar epileptiform discharges and two typical electroclinical seizures, one with right frontal onset and one that lateralized to the right hemisphere but did not further localize.

Three management strategies were considered by our multidisciplinary epilepsy surgery conference and discussed with the patient and his family: continued medication trials, stereo EEG (sEEG) to further evaluate seizure onset zone, and resection of the hamartoma. The rationale to pursue resection was based on the ineloquent, likely nonfunctional, lesion in the setting of significant concordance of his preoperative workup with right frontal semiology, interictal EEG, ictal EEG, and MRI findings.

**Fig. 2:**
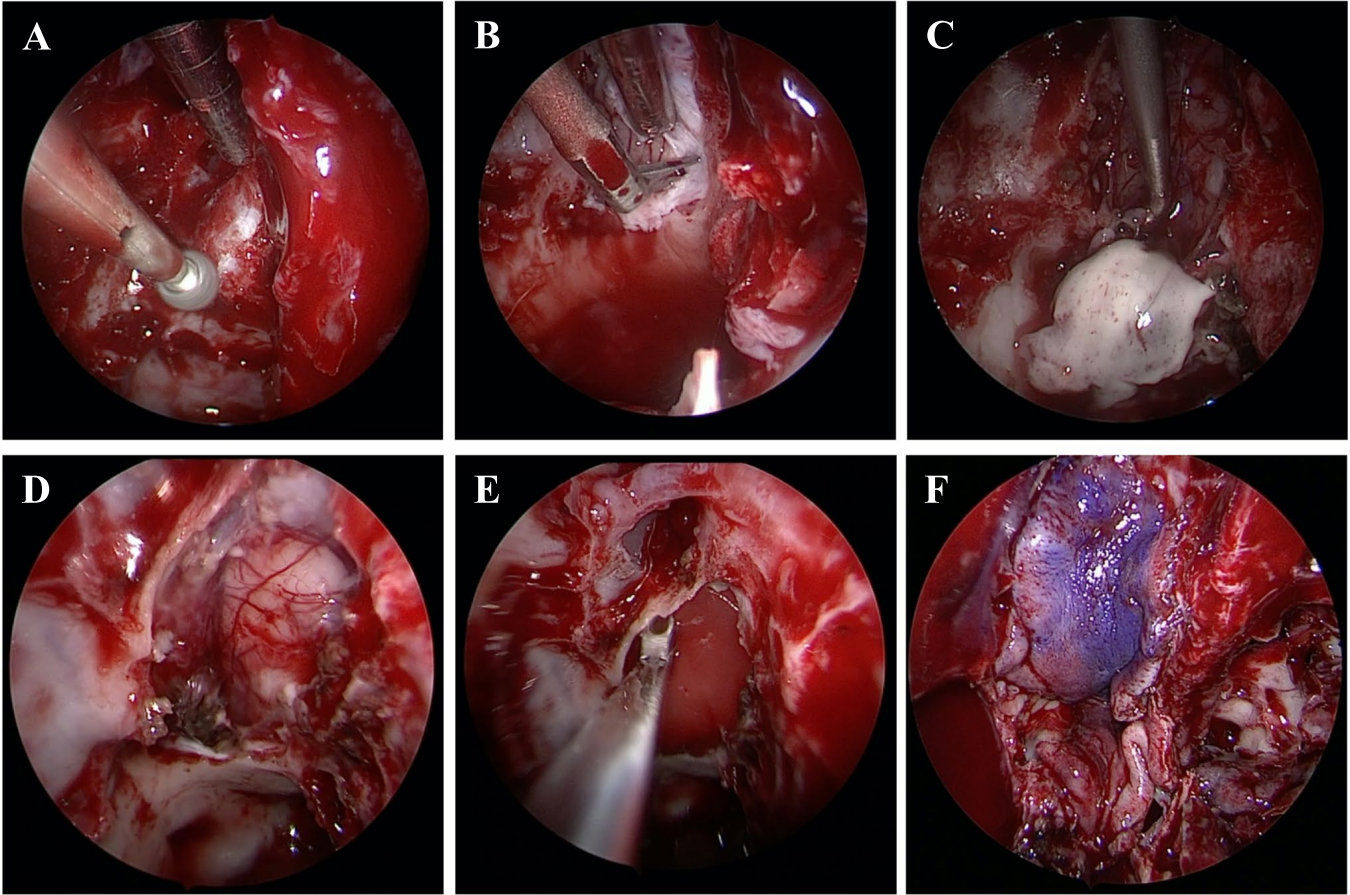
Representative endoscopic intraoperative images showing craniotomy through the cribriform plate (**A**), durotomy (**B**), hamartoma resection (**C**), completed hamartoma resection with maintenance of the suprajacent pial plane (**D**), placement of dural substitute with inlay technique (**E**), and the naso-septal overlay flap in place (**F**)

EETC was pursued rather than craniotomy to avoid all brain retraction, minimize postoperative pain, and optimize cosmesis (Video 1). Consent was obtained for a combined neurosurgery-otorhinolaryngology approach to resection utilizing electromagnetic navigation. A naso-septal flap was raised, and the posterior septum was removed to allow access through both nares. Great care was taken to avoid any contact with the left-sided olfactory epithelium. The right-sided ethmoid sinuses were exposed, and mucosa was removed. The lesion was appreciated by the bony contour, relationship to the anterior and posterior ethmoid arteries, and confirmed with navigation. A high-speed diamond drill bit was used to expose the dura (Fig. 2A). Anterior and posterior ethmoid arteries were cauterized and divided to provide access to the entire lesion. Dura was opened over the lesion in a C-shaped fashion based medially with bipolar cautery and a blade (Fig. 2B). Bipolar and ring curettes were used to dissect the lesion from gyrus rectus and orbitofrontal gyrus (Fig. 2C). There was an arachnoid plane between the lesion and the cerebral cortex. The lesion was firm and the color was consistent with gray matter. The anterior and middle components of the lesion were resected and sent to pathology. The posterior aspect of the lesion was resected until it tapered to the size of a normal tract and appeared to be only white matter (Fig. 2D). Dural substitute was placed inside the durotomy (Fig. 2E) and the naso-septal flap was used as an overlay along with fibrin glue, temporary packing, and nasal trumpets (Fig. 2F).

**Fig. 3:**
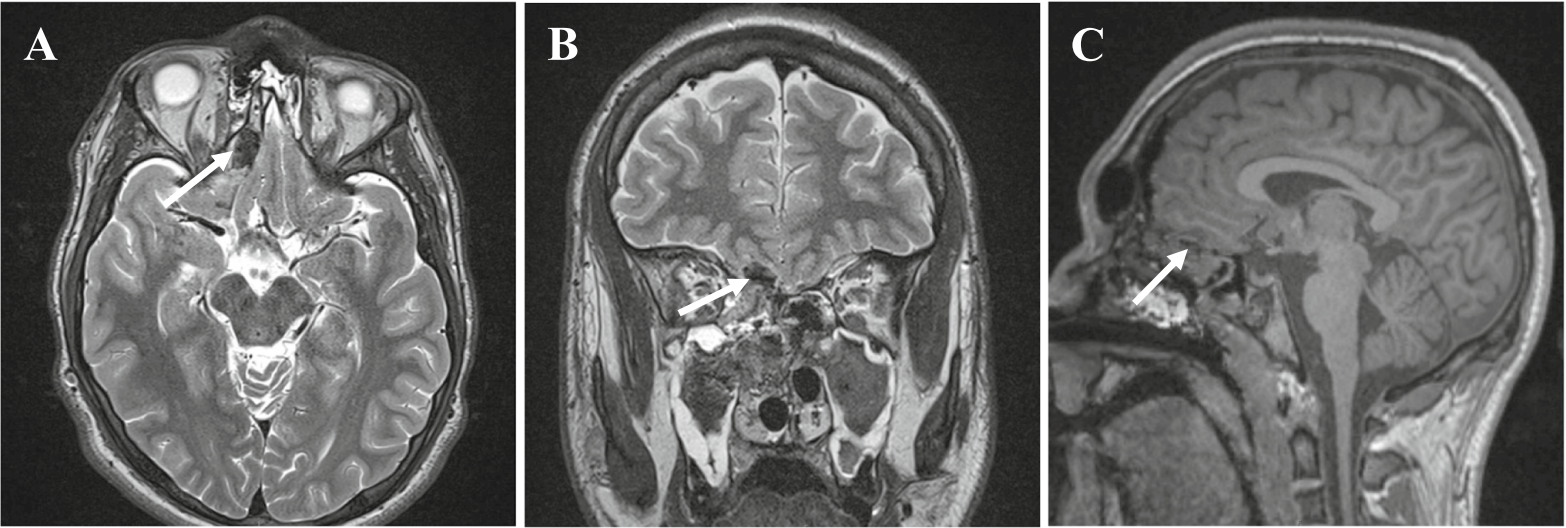
Axial (**A**) and coronal (**B**) postoperative T2-weighted and sagittal postoperative T1-weighted non-contrast (**C**) magnetic resonance images (MRI) obtained the day following surgery showing a gross total resection with otherwise expected post-surgical changes (white arrows)

Post-operative MRI revealed a gross total resection of the lesion (Fig. 3A–C). Histopathology of the sample revealed a collection of non-giant, non-dysmorphic, and singly nucleated medium-sized neurons that did conform to the laminar organization normally observed in the olfactory bulb. While there are no standard criteria for the histologic appearance of olfactory bulb hamartomas, these findings closely resembled the morphology of a hypothalamic hamartoma; and thus, the diagnosis of a hamartoma was made.

The patient’s postoperative course was uncomplicated. Per our standard protocol, his trumpets were removed on post-operative day 2 (POD2). He was discharged home on POD4. At 2-week follow-up, standard in-office endoscopy revealed a well healing flap, and packing was removed. At 6-month follow-up, the patient was doing well and had been seizure-free since surgery (Engel class 1 outcome). There was no concern for cerebrospinal fluid leak, and his neurological exam, notably his olfaction, remained intact.

## Historical background

Glioneuronal hamartomas are benign, non-progressive congenital lesions that infrequently occur. Hypothalamic hamartomas, at an incidence of 1/100,000 [17], and cortical tubers of tuberous sclerosis, at an incidence of 1/60,000 [18], are the most common. Sporadic hamartomas of the cerebral cortex are exceedingly rare [15, 17, 19]. Of the three prior cases of olfactory bulb hamartomas found in the literature, two were in patients with underlying tuberous sclerosis [10, 11]. The remarkable rarity of these lesions has limited our understanding and awareness of these entities.

## Clinical presentation

Medically refractory epilepsy is the most common presenting symptom for glioneural hamartomas [15]. In fact, although such lesions are rare, in some series 3% of patients operated on for epilepsy have been found to have an intracranial hamartoma [20, 21]. The inherent epileptogenicity of hamartomas, likely including those within the olfactory apparatus, is poorly understood but is believed to be related to irregularly arranged neurons with aberrant inter-neuronal connections [22], associated microscopic cortical dysplasia [15], and the observation that these lesions harbor clusters of neurons with inherent pacemaker-like activity [15], although the molecular etiology of this phenomenon is not well characterized [23]. Seizures in our patient were initially well controlled with medication until the frequency increased during his adolescent years. This is fairly consistent with other hamartoma reports which often describe increased frequency and severity of seizures, as well as changes in seizure type, over the disease course [15, 19, 24–27]. For instance, patients with hypothalamic hamartomas often present in infancy with gelastic seizures; however, the majority of patients go on to develop disabling focal with impaired awareness or generalized (e.g., tonic–clonic, drop, or atypical) seizures [17, 28, 29]. The reasoning for such seizure evolution despite lack of imaging changes is likely multifactorial; however, it has been suggested that frequent interictal discharges and repeated seizures over prolonged periods strengthen and expand the excitatory neuronal network responsible for seizure activity [30]. Further work assessing the molecular etiology of the hamartoma-related seizures would aid in understanding the complex clinical manifestations of these rare lesions.

## Diagnosis

Computed tomography (CT) or MR imaging is needed for lesion identification. Imaging characteristics are variable, but lesions are often heterogenous with areas of calcification on CT [3–12, 31]. On MRI, glioneural hamartomas are often isointense (T1) or hyperintense (T2) with some heterogeneity, due to areas of fat and calcification, and typically enhance with contrast [3–12, 31]. An official diagnosis, however, can only be made by histopathological analysis, which will show primarily disorganized mature, small-to-medium sized neurons with low Ki67 positivity and often reactive gliosis [10].

Confirming a hamartoma as the source of seizures is an important part of diagnosis and is vital to guiding management recommendations. Workup should include an extensive clinical history, genetic testing, scalp EEG, and possible intracranial sEEG [3–12, 31].

## Management and outcomes

Epilepsy associated with glioneuronal hamartomas, irrespective of location, is rarely well managed by anti-seizure medications (ASMs) alone. If a proper diagnosis and treatment plan is not made early in the disease course, it can have devastating complications. For instance, nearly 50% of patients with hypothalamic hamartomas develop some type of developmental or intellectual delay, cognitive decline, or psychiatric disorder [19]. While lesion location may play a role in these clinical sequelae, prolonged uncontrollable seizures are likely the main contributor to neurodevelopmental disability in many cases. This hypothesis is consistent with the reported risk factors for cognitive impairment in patients with glioneuronal hamartomas, which includes younger age of seizure onset, higher seizure frequency, a greater number of ASMs used, and more severe seizure subtypes (e.g., tonic–clonic, drop attacks) [17]. Further, while our patient fortunately did not progress to having any developmental disability, his hamartoma may have subjected him to many potentially preventable seizures and hypothyroidism from his ASM therapy. Such considerations emphasize the importance of keeping hamartoma on the differential when a patient presents with epilepsy.

Our patient presented with a prolonged history of focal seizures that increased in frequency over the years despite escalating medical treatment. Although mass effect was mild, the lesion was considered likely to be the source of seizures given the right frontal epileptiform discharges, right frontal onset seizures, left body-predominant hypermotor semiology, and an otherwise negative workup for another epilepsy etiology. This possibility is consistent with the only similar report available in the literature, whereby surgical resection of an olfactory bulb hamartoma led to seizure resolution in a patient with previously intractable epilepsy [10].

The rationale for our treatment approach begins with the decision to operate. Although less-invasive treatment options, such as gamma knife radiosurgery (GRK), stereotactic radiofrequency thermocoagulation (RF-TC), and laser-induced thermal therapy (LiTT) have emerged as safe treatment options for glioneural hamartomas of the hypothalamus [32–35], surgical resection remains the most effective treatment for lesional epilepsy. For hamartomas specifically, surgical resection results in a clinically significant reduction in seizure frequency in 90% of cases, with approximately 70% of patients achieving complete seizure freedom, and was successful in the only other documented case involving a hamartoma of the olfactory apparatus [10, 15, 36, 37]. Furthermore, a selective resection of the hamartoma minimizes the possibility of any intraoperative neurologic injury. Although the EETC approach has not been reported for resection of hamartomas, it provides the most direct access to this lesion without the need for any brain retraction. Additionally, this approach has been associated with lower perioperative morbidity than what is observed for craniotomies done for meningiomas or schwannomas located in the anterior skull base [38–43]. Our experience with endonasal resection of craniopharyngiomas in the pediatric population also supports improved outcomes with low CSF leak rates when compared to open resection [42, 44, 45].

## Conclusions

Our experience with this case demonstrates a unique surgical approach allowing for complete lesional resection with no manipulation of normal brain resulting in minimal patient discomfort, no perioperative complications, and complete seizure freedom. This illustrates the feasibility and advantages of treating this rare lesion, and focal epilepsy, through the EETC approach.

The epileptogenicity of olfactory tract hamartomas is not established, with only one known corroborating case report. Our patient has remained seizure-free since surgery (Engel class 1 outcome), thereby confirming it as the causative lesion for his epilepsy.

The rarity of olfactory tract hamartomas has thus far limited our understanding of these lesions. Research is beginning to highlight aspects of glioneuronal hamartomas that may help us better elucidate the underlying factors that impact their clinical variability. Continued work investigating these lesions, including studying the course of associated epilepsy before and after treatment, will lead to improved individualized treatment strategies.

## Supplementary Information

Below is the link to the electronic supplementary material.

Video 1 (0–6 s) Elevation of the naso-septal flap: the right-sided ethmoid sinuses were exposed, and mucosa was removed. (06–14 s) Performing the craniotomy: a high-speed diamond drill bit was used to expose the dura. (14–22 s) Performing the durotomy: dura was opened over the lesion in a C-shaped fashion based medially with bipolar cautery and a blade (22–46 s) resection of the lesion: bipolar and ring curettes were used to dissect the lesion from gyrus rectus and orbitofrontal gyrus. There was an arachnoid plane between the lesion and the cerebral cortex. The lesion was firm, and the color was consistent with gray matter (46–55 s). Placing the dural substitute inlay; (55–60 s) placing the naso-septal flap (MOV 17166 KB)

## Data Availability

No datasets were generated or analysed during the current study.
